# A CRISPR activation screen identifies genes that enhance SARS-CoV-2 infection

**DOI:** 10.1093/procel/pwac036

**Published:** 2022-08-23

**Authors:** Fei Feng, Yunkai Zhu, Yanlong Ma, Yuyan Wang, Yin Yu, Xinran Sun, Yuanlin Song, Zhugui Shao, Xinxin Huang, Ying Liao, Jingyun Ma, Yuping He, Mingyuan Wang, Longhai Tang, Yaowei Huang, Jincun Zhao, Qiang Ding, Youhua Xie, Qiliang Cai, Hui Xiao, Chun Li, Zhenghong Yuan, Rong Zhang

**Affiliations:** Key Laboratory of Medical Molecular Virology (MOE/NHC/CAMS), School of Basic Medical Sciences, Shanghai Medical College, Shanghai Institute of Infectious Disease and Biosecurity, Fudan University, Shanghai 200032, China; Key Laboratory of Medical Molecular Virology (MOE/NHC/CAMS), School of Basic Medical Sciences, Shanghai Medical College, Shanghai Institute of Infectious Disease and Biosecurity, Fudan University, Shanghai 200032, China; Key Laboratory of Medical Molecular Virology (MOE/NHC/CAMS), School of Basic Medical Sciences, Shanghai Medical College, Shanghai Institute of Infectious Disease and Biosecurity, Fudan University, Shanghai 200032, China; Key Laboratory of Medical Molecular Virology (MOE/NHC/CAMS), School of Basic Medical Sciences, Shanghai Medical College, Shanghai Institute of Infectious Disease and Biosecurity, Fudan University, Shanghai 200032, China; Key Laboratory of Medical Molecular Virology (MOE/NHC/CAMS), School of Basic Medical Sciences, Shanghai Medical College, Shanghai Institute of Infectious Disease and Biosecurity, Fudan University, Shanghai 200032, China; Key Laboratory of Medical Molecular Virology (MOE/NHC/CAMS), School of Basic Medical Sciences, Shanghai Medical College, Shanghai Institute of Infectious Disease and Biosecurity, Fudan University, Shanghai 200032, China; Department of Pulmonary Medicine, Zhongshan Hospital, Shanghai Key Laboratory of Lung Inflammation and Injury, Fudan University, Shanghai 200032, China; The Center for Microbes, Development and Health, CAS Key Laboratory of Molecular Virology & Immunology, Institute Pasteur of Shanghai, CAS Center for Excellence in Molecular Cell Science, University of Chinese Academy of Sciences, Chinese Academy of Sciences, Shanghai 200031, China; Technical Center for Animal, Plant and Food Inspection and Quarantine of Shanghai Customs, Shanghai 200032, China; Shanghai Veterinary Research Institute, CAAS, Shanghai 200241, China; College of Animal Science, South China Agricultural University, Guangzhou 510642, China; Shanghai International Travel Healthcare Center, Shanghai 200335, China; Suzhou Blood Center, Suzhou 215006, China; Suzhou Blood Center, Suzhou 215006, China; Key Laboratory of Animal Virology of Ministry of Agriculture, College of Animal Sciences, Zhejiang University, Hangzhou 310058, China; State Key Laboratory of Respiratory Disease, National Clinical Research Center for Respiratory Disease, Guangzhou Institute of Respiratory Health, the First Affiliated Hospital of Guangzhou Medical University, Guangzhou 510182, China; Center for Infectious Disease Research, School of Medicine, Tsinghua University, Beijing 100086, China; Key Laboratory of Medical Molecular Virology (MOE/NHC/CAMS), School of Basic Medical Sciences, Shanghai Medical College, Shanghai Institute of Infectious Disease and Biosecurity, Fudan University, Shanghai 200032, China; Key Laboratory of Medical Molecular Virology (MOE/NHC/CAMS), School of Basic Medical Sciences, Shanghai Medical College, Shanghai Institute of Infectious Disease and Biosecurity, Fudan University, Shanghai 200032, China; The Center for Microbes, Development and Health, CAS Key Laboratory of Molecular Virology & Immunology, Institute Pasteur of Shanghai, CAS Center for Excellence in Molecular Cell Science, University of Chinese Academy of Sciences, Chinese Academy of Sciences, Shanghai 200031, China; Department of Pulmonary Medicine, Zhongshan Hospital, Shanghai Key Laboratory of Lung Inflammation and Injury, Fudan University, Shanghai 200032, China; Key Laboratory of Medical Molecular Virology (MOE/NHC/CAMS), School of Basic Medical Sciences, Shanghai Medical College, Shanghai Institute of Infectious Disease and Biosecurity, Fudan University, Shanghai 200032, China; Key Laboratory of Medical Molecular Virology (MOE/NHC/CAMS), School of Basic Medical Sciences, Shanghai Medical College, Shanghai Institute of Infectious Disease and Biosecurity, Fudan University, Shanghai 200032, China

**Dear Editor,**

Identifying the host factors that are utilized for virus infection and mapping their cell-type expression profile can help to understand the viral tissue/organ tropism and pathogenesis. Much effort has been devoted to the identification of SARS-CoV-2 infection-dependent host factors. CRISPR-based activation (Konermann et al., 2015), the up-expression of a virus-dependent host gene through the recruitment of specific transcriptional activators to the promoter, as a gain of function strategy, may facilitate the virus infection. In this study, we performed the genome-scale CRISPR activation screen, as a complementary method to previous knockout screens, for SARS-CoV-2 in HeLa cells that express a minimal level of receptor ACE2. The cell library was infected with single-round virus replication particles SARS-CoV-2 GFP/ΔN trVLP (Ju et al., 2021), and the GFP positive cells were sorted for genomic DNA extraction, sgRNA sequencing, and data analysis (Table S1). The top candidates from the screen were determined according to their MAGeCK score (Fig. 1A). In addition to the critically important cellular receptor ACE2 and serine protease TMPRSS2, some other host factors that are known for promoting the SARS-CoV-2 infection, such as the DC-SIGN (CD209; Thepaut et al., 2021) and ITGA5 (Integrin Subunit Alpha 5; Bristow et al., 2020; Sigrist et al., 2020; Beddingfield et al., 2021), were identified, confirming the utility of the screening strategy. It was noteworthy that SIGLEC1 (Sialic Acid Binding Ig Like Lectin 1), which belongs to the C-type lectin family, is the most significantly enriched host gene. Gene ontology enrichment analysis of the top 100 genes revealed that most of the genes were related to virus life cycle, especially viral entry or receptors (Fig. S1). Some genes that regulate the metabolic response were also identified (Fig. S1).

**Figure 1.**
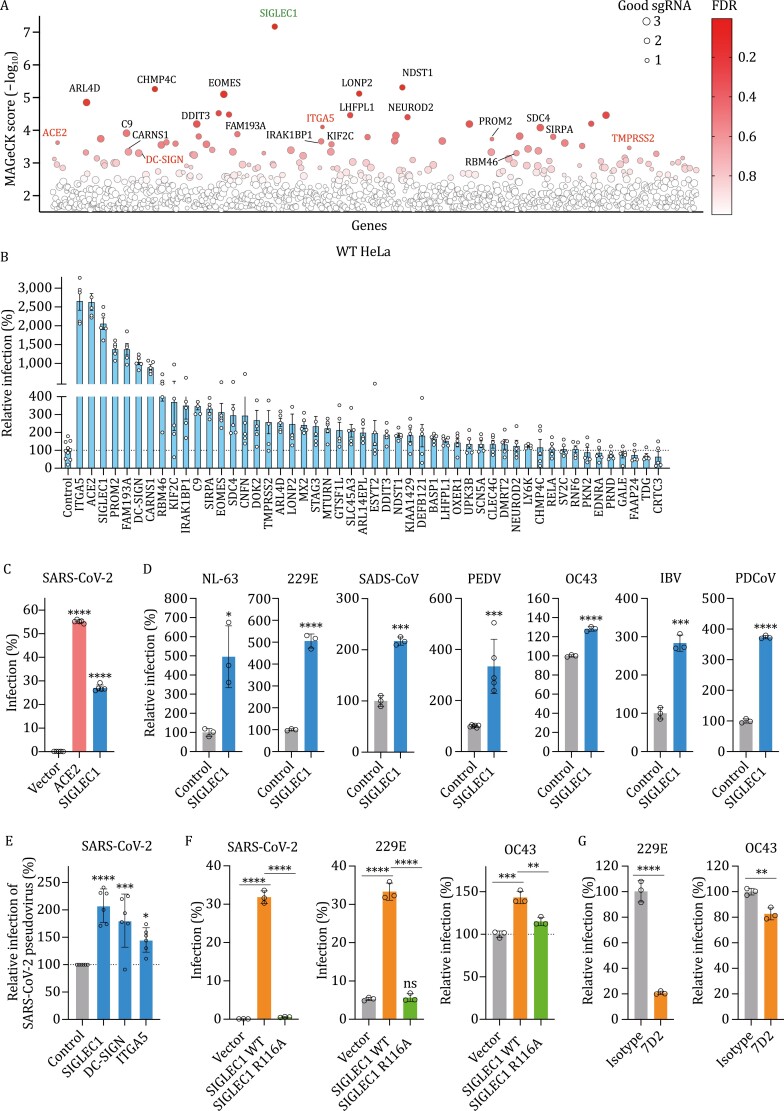
Genome-scale CRISPR activation screen identifies genes that promote SARS-CoV-2 infection. (A) Genes identified from the CRISPR screen. The genes were indicated based on the MAGeCK score and false discovery rate. The size of circle shows the different number of good sgRNAs enriched from the screen. (B) Experimental validation of the top 50 genes. Gene expression was activated using sgRNA in WT HeLa cells that were infected with SARS-CoV-2 virus. (C) cDNA expression and validation by SARS-CoV-2 infection. The human ACE2 and SIGLEC1 cDNA were overexpressed in WT HeLa cells for the experiment. (D) Validation of SIGLEC1 by other coronaviruses. CRISPR sgRNA activated WT HeLa cells were infected with coronaviruses from the genus alpha, beta, gamma, and delta. (E) Pseudovirus infection assay. The expression of selected genes was activated for the infection by pseudovirus bearing the spike protein of SARS-CoV-2. (F) The impact of SIGLEC1 mutation on coronavirus infection. WT SIGLEC1 or R116A mutant were overexpressed in WT HeLa cells and infected with three different coronaviruses. (G) Antibody blockade in HeLa-SIGLEC1 cells.

Validation experiments using individual sgRNAs to activate the endogenous protein expression indicated that ITGA5, ACE2, and SIGLEC1 increase the SARS-CoV-2 infection over 20-fold when compared to the control cells (Fig. 1B). The upregulation of surface SIGLEC1 protein and cellular mRNA was confirmed by flow cytometry and RT-qPCR respectively (Fig. S2A and S2B). Besides the known DC-SIGN, three novel factors, cholesterol-binding protein PROM2 (Prominin 2; Florek et al., 2007), ATPase CARNS1 (ATPGD1, Carnosine Synthase 1; Drozak et al., 2010), and FAM193A (Family With Sequence Similarity 193 Member A) with no clear biological functions, also significantly enhanced the virus infection (Fig. 1B).

As the SIGLEC1 is the top hit on our screen, and to further verify if the SIGLEC1 promotes the SARS-CoV-2 infection, we overexpressed the cDNA of SIGLEC1 in non-permissive HeLa cells, and infected with the virus for nucleocapsid (N) protein expression analysis. As expected, the empty vector control cells showed minimal infection (Fig. 1C). While the ACE2 overexpression conferred the cells to be permissive and over 50% of cells were positive for N protein, the expression of SIGLEC1 promoted the virus infection to around 25% (Fig. 1C). To examine if the SIGLEC1 could be a host proviral factor for other members of coronavirus, we infected endogenously activated HeLa cells with coronaviruses from all four genera, the alpha genus including the HCoV-NL63, HCoV-229E, swine acute diarrhea syndrome coronavirus (SADS-CoV), and porcine epidemic diarrhea virus, the beta genus including HCoV-OC43, the gamma genus including the infectious bronchitis virus, and the delta genus including the porcine deltacoronavirus (PDCoV). The results indicated that SIGLEC1 expression could significantly enhance the infection by all the coronaviruses tested (Fig. 1D). Moreover, the enhancement of virus infection by SIGLEC1 expression was confirmed by using the retrovirus-based pseudovirus bearing the SARS-CoV-2 spike protein (Fig. 1E). The expression of DC-SIGN or ITGA5 also promoted the pseudovirus infection to a variable extent (Fig. 1E).

SIGLEC1 has been shown to facilitate the HIV-1 infection by binding to the ganglioside embedded in the lipid of virions, and the mutation of residue arginine at the position 116 to alanine can abrogate the binding property (Izquierdo-Useros et al., 2012; Puryear et al., 2013). To determine the specificity of SIGLEC1-mediated binding to the ganglioside to promote the virus infection, we overexpressed the wild-type SIGLEC1 or R116A mutant in HeLa cells, and infected with SARS-CoV-2, HCoV-229E, or HCoV-OC43. The surface expression of SIGLEC1 was confirmed (Fig. S2C). The mutation of R116A could obviously decrease the infection by all these three viruses (Fig. 1F). In addition, it is previously reported that antibody blockade such as the clone 7D2 can prevent the binding of SIGLEC1 to the ganglioside as reported for HIV-1 and ebolavirus (Izquierdo-Useros et al., 2012; Puryear et al., 2013; Perez-Zsolt et al., 2019). Likewise, we found that pre-incubation of SIGLEC1 blocking antibody in HeLa-SIGLEC1 cells could significantly decrease the infection by both HCoV-229E and HCoV-OC43 (Fig. 1G).

Moreover, to assess if SIGLEC1 facilitating virus infection is mediated by the primary receptor ACE2, three ACE2-deficient cell lines, HeLa, A549, and 293T, were employed. It showed that overexpression of SIGLEC1 in ACE2-knockout 293T or ACE2-knockout A549 does not facilitate the SARS-CoV-2 infection (Fig. S3). However, overexpression of SIGLEC1 in ACE2-knockout HeLa still promotes the infection, albeit much less efficient than that in wild-type HeLa cells (Fig. S3), suggesting that the role of SIGLEC1 in promoting SARS-CoV-2 infection primarily depends on the receptor ACE2. In some cell types such as HeLa, other known or unknown candidate receptors instead of ACE2 may coordinate with SIGLEC1 to enhance the virus infection.

SIGLEC1 is highly expressed on mature DCs and mediates the trans-infection of HIV-1 to CD4^+^ T cells (Izquierdo-Useros et al., 2012). The expression of SIGLEC1 on DCs is significantly upregulated by interferon or lipopolysaccharide (LPS) stimulation (Izquierdo-Useros et al., 2012; Puryear et al., 2013). To investigate the role of SIGLEC1 on DCs in SARS-CoV-2 infection, we prepared the monocyte-differentiated immature DCs and treated with IFN-α or LPS, resulting in the maturation of DCs. The purity of DCs was analyzed by detecting the surface expression of markers (Fig. S4). As expected, the expression level of SIGLEC1 was increased in mature DCs (Fig. S5A), while the DC-SIGN was constitutively expressed and not changed (Fig. S5B). The binding of SARS-CoV-2 virions on mature DCs was slightly increased, especially on the LPS-stimulated DCs (Fig. 2A). To examine if the virion internalization is enhanced in mature DCs, cells were pre-incubated with SIGLEC1 blocking antibody 7D2, and then incubated with the virions for 1 or 4 h. The internalized virions bearing the genomic RNA were quantified by qRT-PCR. The virus internalization was significantly prevented by blocking antibody pretreatment when compared to the isotype control (Fig. 2B). The addition of mannan, a C-type lectin inhibitor to block the DC-SIGN (Izquierdo-Useros et al., 2012), had a synergistic effect with the blocking antibody (Fig. 2B). The blocking effect on virion internalization was further confirmed by detecting the minus-strand viral RNA, an indicator of the viral replication initiated (Fig. 2C). Moreover, the blocking effect of SIGLEC1-mediated virion entry by antibody was diminished when the virus stock was prepared in the presence of chemical reagent PDMP, a competitive inhibitor of glycosylceramind synthase (Fig. 2D) (Hatch et al., 2009). These results suggested that SIGLEC1 can enhance SARS-CoV-2 infection in mature DCs.

**Figure 2.**
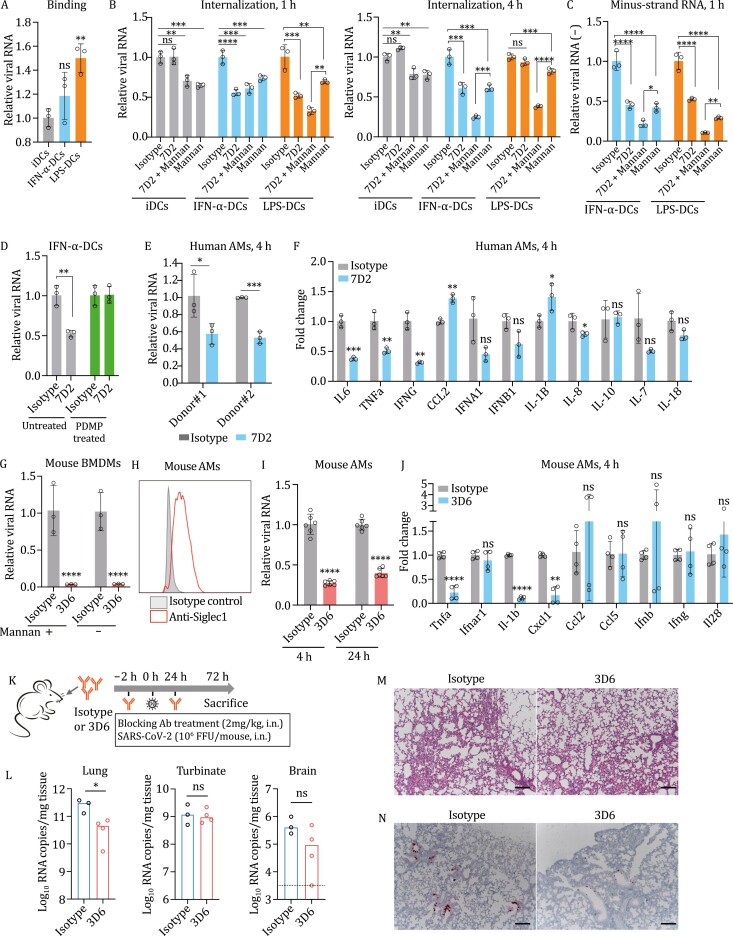
Blockade of SIGLEC1 reduces SARS-CoV-2 infection in primary cells and in human ACE2 knock-in mice. (A) Virion binding assay in different DCs. (B) Blockade of SIGLEC1 by antibody decreases virion internalization. The DCs were pretreated with isotype or SIGLEC1 blocking antibody 7D2 in the presence or absence DC-SIGN inhibitor mannan, the internalized virions were quantified by qRT-PCR at 1 or 4 h. (C) Detection of minus-strand RNA synthesis at 1 h for the internalized virion by qRT-PCR. (D) The effect of glucosylceramide synthase competitive inhibitor on virion internalization. The virion stock was prepared in Calu-3 cells with or without PDMP treatment, then used for internalization assay in IFN-α-DCs. The surface SIGLEC1 was blocked with isotype or specific antibody clone 7D2. (E) Antibody blockade decreases virus infection in hAMs. The hAMs were pretreated with isotype or blocking Ab 7D2, and viral RNA in cells at 4-hour post-infection was quantified by qRT-PCR. (F) Antibody blockade reduces the virus-induced cytokine production in hAMs. The hAMs were pretreated with isotype or blocking Ab 7D2, and the mRNA expression levels for the indicated cellular cytokines at 4-hour post-infection were quantified by qRT-PCR. (G) Virion infection in mouse bone marrow–derived macrophages (BMDMs). Mouse BMDMs were pretreated with isotype or SIGLEC1 blocking antibody 3D6 in the presence or absence of DC-SIGN inhibitor mannan, viral RNA in cells was quantified at 1-hour post-infection. (H) Surface expression of SIGLEC1 in mouse primary alveolar macrophages. (I) Antibody blockade decreases virus infection in mouse AMs. The mAMs were pretreated with isotype or blocking Ab 3D6, and viral RNA in cells at 4- and 24-hour post-infection was quantified by qRT-PCR. (J) Antibody blockade reduces the virus-induced cytokine production in mAMs. The mAMs were pretreated with isotype or blocking Ab 3D6, and the mRNA expression levels for the indicated cellular cytokines at 24-hour post-infection were quantified by qRT-PCR. (K) Scheme of antibody treatment and SARS-CoV-2 challenge. (L) Viral loads in the tissues of lung, nasal turbinate, and brain. The human ACE2 knock-in mice were treated with isotype or SIGLEC1 blocking antibody 3D6. Tissues were harvested at day 3 post-challenge of SARS-CoV-2 to quantify the viral RNA by qRT-PCR. (M) H&E staining of lung sections of challenged mice. Representative images are shown from *n* = 3 mice. Scale bar, 100 μm. (N) RNA ISH of lung sections of challenged mice. Representative images are shown from *n* = 3 mice. Scale bar, 100 μm.

Macrophages play significant roles in virus infection and inflammation. Resident alveolar macrophages (AMs) with SIGLEC1 expression are present with high frequency in the BAL samples compared to digested lung tissues (Bharat et al., 2016; Yu et al., 2016). To investigate if SIGLEC1 could enhance the infection of SARS-CoV-2 in AMs, we isolated the primary AMs from human BAL fluid with noninflammatory or noninfectious disorders. Antibody blockade with 7D2 followed by virus infection significantly reduced the viral RNA at both 4 and 24 h (Figs. 2E and S6A). The differential cytokine production was also determined. Antibody blockade could obviously reduce the cytokine mRNA levels such as *IL-6*, *TNFa*, *IFNG*, *IFNA1*, *IFNB1*, at 4- or 24-hour post-infection (Figs. 2F and S6B).

To rule out the possible influence of cellular receptor ACE2 on virus infection in human primary AMs, we switched to the mouse model in which the ACE2 does not support the wild-type SARS-CoV-2 entry. We first tested the bone marrow–derived macrophages and found that blocking the mouse SIGLEC1 with specific antibody 3D6 (Erikson et al., 2015) can markedly inhibit the virus infection (Fig. 2G). Then, we isolated the mouse primary AMs, and the surface expression of mouse SIGLEC11 was confirmed (Fig. 2H). Similarly, antibody blockade by 3D6 obviously prevented the virus infection at 4 and 24 h (Fig. 2I). In addition, the expression of cytokine mRNA levels such as *Tnfa*, *Il-1b*, and *Cxcl1* was significantly reduced (Fig. 2J). These results from both human and mouse primary AMs suggested that SIGLEC1 plays an important role in promoting virus infection and cytokine production.

Since expression of SIGLEC1 is present on AMs, and blockade of SIGLEC1 *ex vivo* significantly reduced the SARS-CoV-2 infection, it would be intriguing to investigate if SIGLEC1 could promote virus infection *in vivo*, especially in the lung where AMs are abundant. We administered human ACE2 knock-in mice intranasally with SIGLEC1 blocking antibody 3D6 or isotype, followed by intranasal inoculation of SARS-CoV-2. The antibodies were given again at 24-hour post-infection, and mice were sacrificed on day 3 for tissue harvesting (Fig. 2K). Although no difference in viral load was observed in the turbinate representing the upper respiratory tract, the viral RNA in lungs treated with SIGLEC1 blocking antibody was slightly reduced (Fig. 2L). The viral RNA in the brain had the trend to decrease when given the blocking antibody, although not statistically significant (Fig. 2L). We could not see the difference in histopathological changes by H&E staining in lungs (Fig. 2M), but RNA ISH indicated that viral RNA appears to be less in the lungs of mice treated with blocking antibody than that with isotype (Fig. 2N). Thus, blockade of the SIGLEC1 could potentially reduce the virus infection in the lung where the AMs are abundant.

In this study, we employ the genome-scale CRISPR-based recruitment of transcription factors to upregulate the expression of endogenous genes and identify the lectin proteins, such as SIGLEC1, that promote the pan-coronavirus infection. The identification of host factors mediating the infection is of significance to understand the tissue tropism and pathogenesis of SARS-CoV-2.

## Supplementary data

The authors declare that all relevant data supporting the findings of this study are available within the paper and its Supplementary information. The Supplemental Data provide information for the CRISPR-Cas9 screen and statistical analysis.

pwac036_suppl_Supplementary_Material

pwac036_suppl_Supplementary_Table_S1

pwac036_suppl_Supplementary_Table_S2

## Footnotes

We wish to acknowledge colleagues at the Biosafety Level 3 Laboratory of Fudan University for help with the technical assistance.

The authors declare no competing interests.

Grants from the National Key Research and Development Program of China (2020YFA0707701 to R.Z.), National Natural Science Foundation of China (32041005 to R.Z.), Project supported by Shanghai Municipal Science and Technology Major Project, Natural Science Foundation of Shanghai (19ZR1470400 to X.H.), Science and Technology Commission of Shanghai Municipality (20DZ2261200), Development Programs for COVID-19 of Shanghai Science and Technology Commission (20431900401), Project of Novel Coronavirus Research of Fudan University (to Y.X.), The Health Science and Technology Program of Suzhou (GWZX202004), and Suzhou Medical Key Discipline Funding Project (SZXK202118) supported this work.

All procedures followed were in accordance with the ethical standards of the Medical Ethical Council of Zhongshan Hospital (B2017-122).

Informed consent was obtained from all individual participants included in the study.

The data underlying this article are available upon request.

F.F., Y.Z., Y.M., Y.W., Y.Y., X.S., C.L., Z.S., R.Z. performed the experiments. F.F., Y.Z., Y.M., Y.W., R.Z. designed the experiments. J.M., Y.L., Y.He., Y.Huang., J.Z., Q.D., Y.X., Q.C., X.H., H.X., Y.S., Z.Y., M.W., L.T. provided administrative, supervision, technical, or material support. F.F., Y.Z., Y.M., R.Z. performed data analysis. R.Z. wrote the initial draft of the manuscript, with the other authors contributing to editing into the final form.
