# Supplementary material for: A CRISPR activation screen identifies genes that enhance SARS-CoV-2 infection
Source: Protein Cell. 2022 Aug 23;14(1):64–8. doi: 10.1093/procel/pwac036 (PMC9871949; doi:10.1093/procel/pwac036)
Supplement: pwac036_suppl_Supplementary_Material [file pwac036_suppl_supplementary_material.docx]

**A CRISPR activation screen identifies genes that enhance SARS-CoV-2 infection**

Fei Feng^1§^, Yunkai Zhu^1§^, Yanlong Ma^1§^, Yuyan Wang^1§^, Yin Yu^1^, Xinran Sun^1^, Yuanlin Song^2^, Zhugui Shao^3^, Xinxin Huang^4^, Ying Liao^5^, Jingyun Ma^6^, Yuping He^7^, Mingyuan Wang^8^, Longhai Tang^8^, Yaowei Huang^9^, Jincun Zhao^10^, Qiang Ding^11^, Youhua Xie^1^, Qiliang Cai^1^, Hui Xiao^3^, Chun Li^2^*, Zhenghong Yuan^1^*, and Rong Zhang^1^*

**Materials and methods**

**Cells and viruses.** Vero E6 (Cell Bank of the Chinese Academy of Sciences, Shanghai, China), HEK 293T (ATCC # CRL-3216), HeLa (ATCC #CCL-2), HeLa-ACE2(Zhu et al., 2021), Huh7, swine testicular (ST), LLC-MK2, and HRT-18, all were cultured at 37°C in Dulbecco’s Modified Eagle Medium (Hyclone #SH30243.01) supplemented with 10% fetal bovine serum (FBS), 10 mM HEPES, 1 mM Sodium pyruvate, 1× non-essential amino acids, and 100 U/ml of Penicillin-Streptomycin. All cell lines were tested routinely and free of mycoplasma contamination. The SARS-CoV-2 (nCoV-SH01-Sfull) stock(Zhu et al., 2021), was prepared in Calu-3 cells. To generate the ganglioside-depleted virus particles, Calu-3 cells were pretreated for 24 h with 10uM D-threo-1-phenyl-2-decanoylamino-3-morpholino-1-propanol (PDMP), a glucosylceramide synthase competitive inhibitor(Hatch et al., 2009), then infected with SARS-CoV-2 for around 48 h in the presence of PDMP, and the supernatant were clarified and aliquot for the experiments.

Swine acute diarrhea syndrome coronavirus (SADS-CoV), porcine epidemic diarrhea virus (PEDV), and infectious bronchitis virus (IBV) were propagated in Vero E6 cells and titrated in the Vero E6 by focus-forming assay(Pal et al., 2013). Other virus stock of coronaviruses, porcine deltacoronavirus (PDCoV) (ST cells), 229E (Huh7 cells), NL63 (LLC-MK2 cells), OC43 (HRT-18 cells), were prepared and titrated similarly in their respective cell lines. All experiments involving SARS-CoV-2 live virus infection were performed in the biosafety level 3 (BSL-3) facility of Fudan University following the regulations.

**Genome-wide CRISPR sgRNA screen.** The human CRISPRa sgRNA Calabrese pooled library Set A targeting 18,885 genes was a gift from David Root and John Doench (Addgene #92379) and packaged in 293FT cells after co-transfection with psPAX2 (Addgene #12260) and pMD2.G (Addgene #12259) at a ratio of 2:2:1 using Fugene®HD (Promega). At 48 h post transfection, supernatants were harvested, clarified by spinning at 3,000 rpm for 15 min, and stored at -80°C. For the CRISPR sgRNA screen, HeLa-dCas9-VP64 cells were generated by transduction of WT HeLa cells with a packaged lentivirus derived from the lenti dCAS-VP64_Blast (Addgene #61425). The HeLa-dCas9-VP64 cells were transduced with packaged sgRNA lentivirus library at a multiplicity of infection (MOI) of ~0.3 by spinoculation at 1000g and 32°C for 30 min in 12-well plates. After selection with puromycin for around 7 days, cells were inoculated with SARS-CoV-2 GFP/△N trVLP expressing the GFP(Ju et al., 2021). The cells were then harvested and sorted for the GFP positive population. Genomic DNA from both sorted cells and uninfected cells was extracted for sgRNA amplification and next generation sequencing using an Illumina NovaSeq 6000 platform. The sgRNA sequences targeting specific genes were extracted using the FASTX-Toolkit (http://hannonlab.cshl.edu/fastx_toolkit/) and cutadapt 1.8.1, and further analyzed for sgRNA abundance and gene ranking by a published computational tool (MAGeCK) (see Supplementary table 1).

**Gene validation.** Top 50 genes from the MAGeCK analysis were selected for validation. The highly enriched sgRNAs from the screen were cloned into the plasmid pXPR_502 (Addgene #96923) and packaged with plasmids psPAX2 and pMD2.G. HeLa-dCas9-VP64 or HeLa-ACE2-dCas9-VP64 cells were transduced with lentiviruses expressing individual sgRNA and selected with puromycin for 7 days. The gene-activated bulk cells were infected with members of coronavirus family, and then subjected for immunofluorescence staining, high-content imaging or flow cytometry analysis.

**Pseudotyped virus experiment.** SARS-CoV2 pseudoviruses were packaged as previously described(Zhu et al., 2021). Shortly, pcDNA3.1 expressing the spike gene of SARS-CoV2 lacking the C-terminal 21 amino acids was co-transfected in HEK 293T cells with the retrovector expressing the nanoluciferase gene and plasmid expressing the MLV Gag-Pol using Fugene®HD transfection reagent (Promega). The virus entry was assessed by transduction of pseudoviruses in gene-activated cells in 96-well plates. After 48 h, the luciferase activity was determined using Nano-Glo® Luciferase Assay kit (Promega #N1110) and the luminescence was recorded by using a FlexStation 3 (Molecular Devices).

**Infection in ACE2 knockout cells.** The human *ACE2* sgRNA (5’-ACAGTTTAGACTACAATGAG-3’) was cloned into the plasmid lentiCRISPR v2 and packaged into lentivirus. Wild type HeLa or A549 cells was transduced with the lentivirus for clonal cell isolation. The ACE2 knockout clones were verified by sequencing. The ACE2 knockout 293T cells were obtained from ABclonal (RM02231). Wild type HeLa and three ACE2-knockout cell lines were transduced with lentivirus bearing the human SIGLEC1 or just empty vector. Stable cell lines were infected with SARS-CoV-2 for viral N protein staining, followed by high-content imaging analysis. Alternatively, SARS-CoV-2 infected cells were subjected for RT-qPCR to measure the N gene.

**Bone Marrow-Derived Macrophage preparation.** Bone Marrow–Derived Macrophages (BMDMs) were generated from the hind limbs of 6-week-old C57BL/6 male mice. Bone marrow was differentiated in the presence of 20% L929 cell-conditioned medium and 10% FBS DMEM for 3 days, then half of the medium was removed and refreshed with same medium and cultured for another 3 days. BMDMs were seeded in 96-well or 24-well plate for virus internalization assay as described below.

**Primary alveolar macrophage isolation**. Mouse alveolar macrophages (AMs) were harvested from 8-week-old C57BL/6 male mice. The mice were anaesthetized and a small incision was made on the trachea to allow the insertion of catheter. Ice-cold PBS with 1mM EDTA was used to flush out alveolar macrophages from the lungs. Human AMs were isolated from the bronchoalveolar lavage fluid (BALF). BALF was obtained from routine bronchoscopies for noninflammatory/noninfectious disorders. The retrieved BALF was transferred immediately from the clinic to the laboratory at 4°C and processed by filtering through a cell strainer to remove mucus and particular debris. Both mouse and human AMs were pelleted at 250 × g at 4°C for 10 min, and seeded in 96-well or 24-well plate for use.

**Human monocyte-derived dendritic cell preparation.** CD14 positive monocytes were isolated from human Peripheral Blood Mononuclear Cells (PBMCs) using CD14 positive Selection Cocktail (STEMCELL #17858C) and RapidSpheres^TM^ (STEMCELL #50100) according to the manufacturer’s instructions. Monocytes were cultured in 10% FBS of RPMI 1640 medium supplemented with 250 U/ml human IL-4 (STEMCELL #78045.1) and 1000 U/ml human GM-CSF (STEMCELL #78015.1). The cytokines were refreshed at day 2, and the cells were cultured for another 3 days for down-stream experiments. The purify of differentiated immature dentritic cells (iDCs) was examined by staining with CD45, CD11c, HLA-DR, and DC-SIGN/CD209, together with a cell viability dye. To activate the immature DCs (iDCs), cells were further treated with 100 ng/ml LPS (InvivoGen #tlrl-b51ps) or IFN-α2b (Sigma #SRP4595) for 2 days. iDCs, LPS activated DCs (LPS-DCs), and IFN-α2b activated DCs (IFN-α-DCs) were seeded in 96-well or 24-well plate for binding and internalization assays.

**Virus binding and internalization assays.** Human DCs and human primary AMs were seeded in 96-well plate or 24-well plate one day prior to the assays. Cells were pre-incubated with 20 μg/ml anti-SEGLEC1 (7D2, abcam #ab18619) blocking antibody or isotype control (ThermoFisher #14-4714-85) for 30 min at 37°C, then on ice for 10 min. Ice-cold SARS-CoV-2 virus (MOI of 20) in an equal volume was added and incubated with cells on ice for 1 h. After five cycles of washing, cells were lysed in TRIzol reagent (ThermoFisher #15596018) for RNA extraction. For internalization assay, after pre-incubation with antibodies for 30 min at 37°C, SARS-CoV-2 virus (MOI of 20) was added and incubated at 37°C for 1 h or 4 h. Cells were chilled on ice, washed 5 times with ice-cold PBS, then lysed in TRIzol reagent for RNA extraction. qRT-PCR was conducted to quantify the viral specific nucleocapsid RNA and an internal control ACTB or GAPDH. For mouse BMDMs and primary mouse AMs, similar binding and internalization assays were conducted as described above, except that different blocking anbibodies 3D6 (3D6, biolegend #142402) and isotype (RTK2758, biolegend # 400502) were used.

**Minus-strand RNA analysis**. Human monocyte differentiated DCs were seeded in 96-well plate one day prior to the assay. Cells were pre-incubated with 20 μg/ml anti-SEGLEC1 blocking antibody (7D2) or isotype control for 30 min at 37°C, SARS-CoV-2 virus (MOI of 20) in an equal volume was added and incubated at 37°C for 1 h. Cells were chilled on ice, washed 5 times with ice-cold PBS, then lysed in TRIzol reagent for RNA extraction. Reverse transcription using the forward primer was conducted with the kit, then the cDNA was used for qPCR to quantify the viral specific nucleocapsid RNA. The ACTB was used as an internal control. Strand-specific primer used for reverse transcription and primers used for qRT-PCR are listed in Supplementary table 2.

**Go and KEGG analysis**. Go and KEGG analysis was performed using the OmicShare tools, a free online platform for data analysis (https://www.omicshare.com/tools). The top 100 genes from screening list were selected for analysis.

**Immunofluorescence staining and analysis**. Virus-infected cells in plates were fixed with 4% paraformaldehyde in PBS for 30 min, permeablized with 0.2% Triton X-100 for 1 h. Cells were then incubated with house-made mouse anti-SARS-CoV-2 nucleocapsid protein serum (1:1000) for 2 h at room temperature. After three washes, cells were incubated with the secondary goat anti-mouse IgG (H + L) conjugated with Alexa Fluor 555 (Thermo #A-21424, 2 μg/ml) for 1 h at room temperature, followed by staining with 4’,6-diamidino-2-phenylindole (DAPI). Images were collected using an Operetta High Content Imaging System (PerkinElmer), and processed using the PerkinElmer Harmony high-content analysis software v4.9 and ImageJ v2.0.0 (http://rsb.info.nih.gov/ij/).

For flow cytometry analysis, virus-infected cells were harvested with trypsin, and fixed with 2% paraformaldehyde in PBS for 10 min. Cells were permeablized with 0.1% saponin in PBS for 10 min, and stained with house-made mouse anti-nucleocapsid protein sera for 30 min at room temperature. After washing, cells were incubated with the secondary goat anti-mouse IgG (H + L) conjugated with Alexa Fluor 647 (Thermo #A21235, 2 μg/ml) for 30 min at room temperature. After two additional washes, cells were subjected to flow cytometry analysis (Thermo, Attune™ NxT) and data processing (FlowJo v10.0.7).

For surface staining and analyzed by flow cytometry, cells were collected with TrypLE (Thermo #12605010) and incubated with the mouse anti-human DC-SIGN (Biolegend #330102), mouse anti-human SIGLEC1(7239, abcam #ab199401), or rat anti-mouse SIGLEC1 (3D6, Biolegend #142402) primary antibody at 4 °C for 30 min. After washing, cells were stained with goat anti-mouse IgG (H + L) conjugated with Alexa Fluor 647 (Thermo #A21235, 2 μg/ml) or goat anti-rat IgG (H + L) conjugated with Alexa Fluor 568 (Thermo #A11077, 2 μg/ml) for 30 min at 4 °C and subjected to flow cytometry analysis.

**Animal experiments**. Six week-old human ACE2 knock-in male mice (Gempharmatech #T037630) were used in the study. The knock-in mice were generated in the background of C57BL/6JGpt by replacing the extracellular domain of mouse ACE2 with its corresponding human ACE2 domain. The expression of chimeric ACE2 was driven by the endogenous mouse ACE2 promoter. The experiment protocol has been approved by the Animal Ethics Committee of School of Basic Medical Sciences at Fudan University, and conducted in the BSL-3 laboratory of Fudan University. The mice were administered intranasally with 1.5 mg/ml isotype or 3D6 blocking antibodies in 50 μl 2 h prior to SARS-CoV-2 challenge. 1.5x10^6^ focus-forming unit (FFU) of virus in 50 μl was inoculated intranasally. The same doses of antibodies were also given at day one post infection. Animals were euthanized at day 3 post infection, and the turbinate, lungs, and brain were harvested, homogenized in DMEM for virus titration by RT-qPCR analysis as described below.

**Histology and RNAscope *assay*.** Virus-infected mice were euthanized and lung tissues were harvested and fixed in 4% paraformaldehyde (PFA) for 48 h. Tissues were embedded in paraffin for sectioning and stained with hematoxylin and eosin (H&E) to assess tissue morphology. To determine sites of virus infection, RNA *in situ* hybridization was performed using the RNAscope 2.5 HD Assay (Red Kit) according to the manufacturer’s instructions (Advanced Cell Diagnostics). Briefly, sections were deparaffinized, treated with H_2_O_2_ and Protease Plus prior to probe hybridization. A probe specifically targeting the SARS-CoV-2 spike RNA (Advanced Cell Diagnostics, #848561) was used for *in situ* hybridization (ISH) experiments. Tissues were counterstained with Gill’s hematoxylin. Tissue sections were visualized using a Nikon Eclipse microscope.

**qRT-PCR.** RNA from tissues or cells was extracted with the TRIzol reagent (ThermoFisher #15596018). Viral or host RNA levels were determined using the TaqPath™ 1-Step RT-qPCR Master Mix (ThermoFisher # A15299) on CFX Connect Real-Time System (Bio-Rad) instrument. A standard curve was produced using serial 10-fold dilutions of *in vitro* transcribed RNA of N gene driven by the SP6 promoter (ThermoFisher #AM1340). Viral burden was expressed on a log_10_ scale as viral RNA copies per mg of tissue. Viral or host RNA was also reverse transcribed into cDNA with PrimeScript™ RT Reagent Kit (TaKaRa # RR047A) using RT primer mix of Oligo dT primer and random 6 mers. qRT-PCR was performed using TB Green® Premix Ex Taq™ II (TaKaRa #RR820A) on the CFX Connect Real-Time System (Bio-Rad) instrument. Relative viral RNA or gene expression were calculated as relative to GAPDH or ACTB. Primers used for qRT-PCR are listed in Supplementary table 2.

**Statistical analysis.** Statistical significance was assigned when *P* values were < 0.05 using Prism Version 9 (GraphPad). Data analysis was determined by a ANOVA, unpaired t-test, or Mann-Whitney depending on data distribution and the number of comparison groups.

**References**

Hatch, S.C., Archer, J., and Gummuluru, S. (2009). Glycosphingolipid composition of human immunodeficiency virus type 1 (HIV-1) particles is a crucial determinant for dendritic cell-mediated HIV-1 trans-infection. J Virol 83, 3496-3506.

Ju, X., Zhu, Y., Wang, Y., Li, J., Zhang, J., Gong, M., Ren, W., Li, S., Zhong, J., Zhang, L.*, et al.* (2021). A novel cell culture system modeling the SARS-CoV-2 life cycle. PLoS Pathog 17, e1009439.

Pal, P., Dowd, K.A., Brien, J.D., Edeling, M.A., Gorlatov, S., Johnson, S., Lee, I., Akahata, W., Nabel, G.J., Richter, M.K.*, et al.* (2013). Development of a highly protective combination monoclonal antibody therapy against Chikungunya virus. PLoS Pathog 9, e1003312.

Zhu, Y., Feng, F., Hu, G., Wang, Y., Yu, Y., Zhu, Y., Xu, W., Cai, X., Sun, Z., Han, W.*, et al.* (2021). A genome-wide CRISPR screen identifies host factors that regulate SARS-CoV-2 entry. Nat Commun 12, 961.

**Supplemental figures**


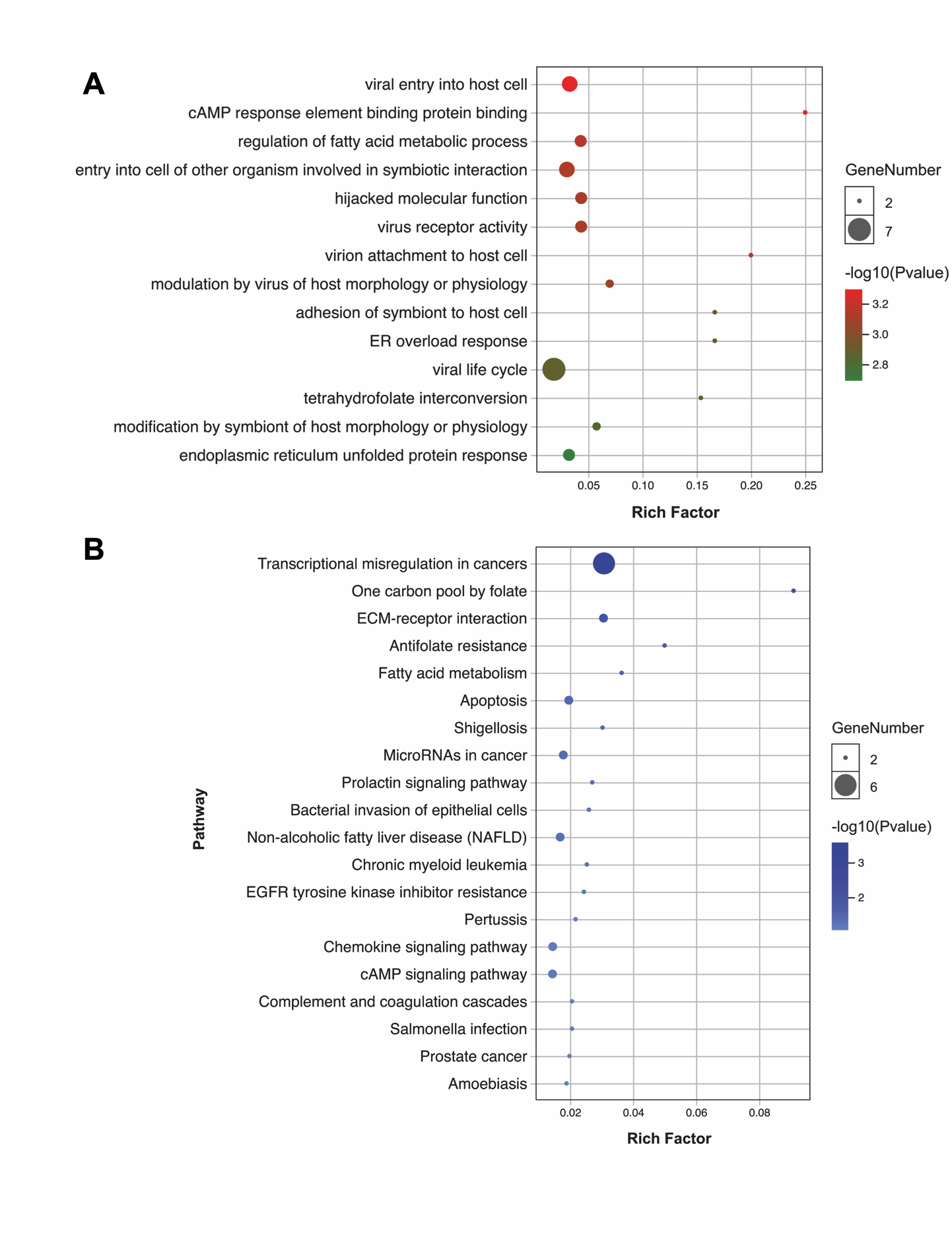


**Fig.S1. Gene Ontology (GO) enrichment and** **KEGG pathway analysis of the genes identified. A.** Gene Ontology (GO) enrichment analysis. **B.** KEGG pathway analysis. The top 100 genes were selected for analysis, and the number of genes identified were shown as different size of circles. The p value was shown in different colors.


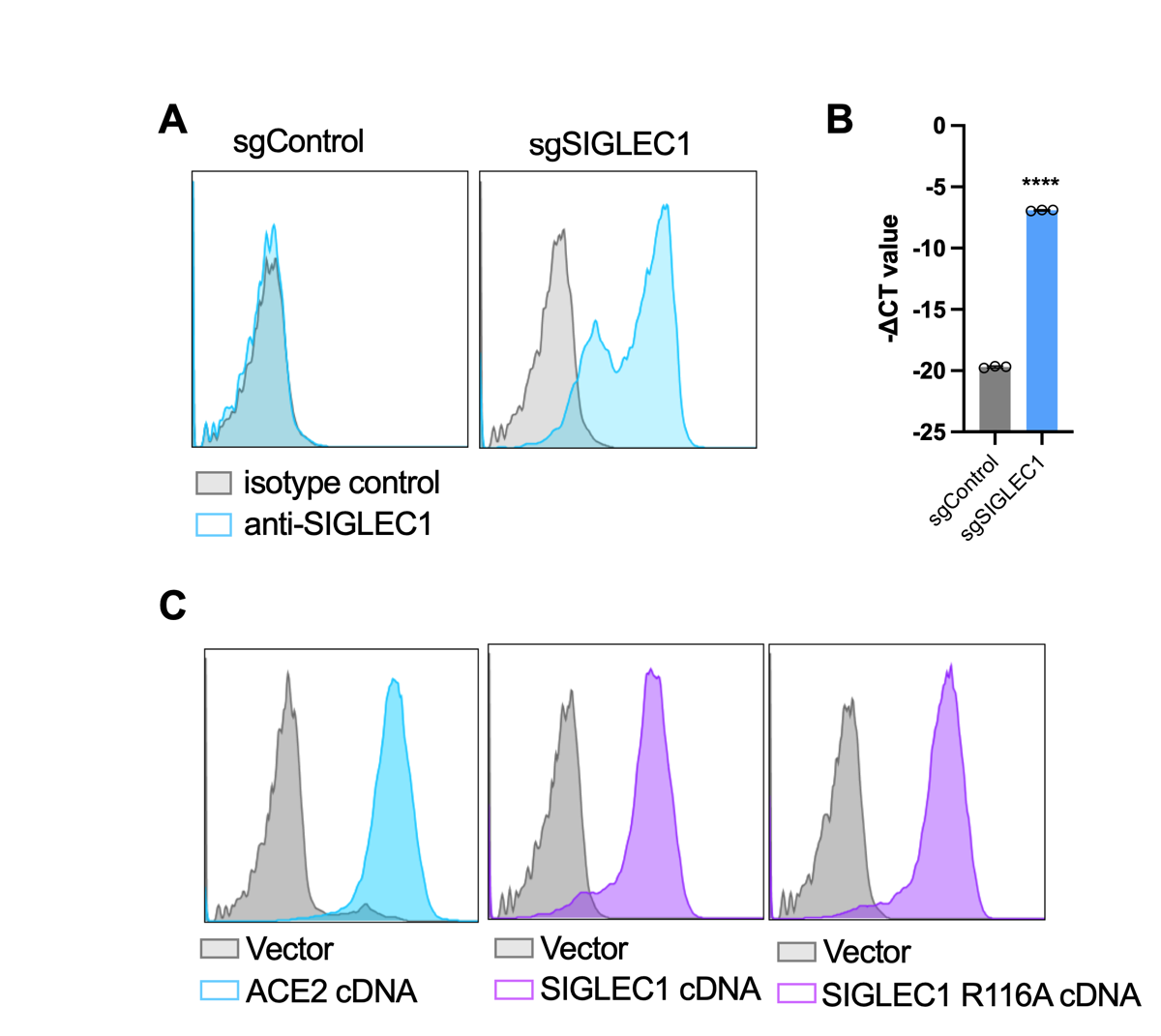


**Fig.S2. Expression of SIGLEC1 activated by CRISPR sgRNA in HeLa cells. A.** Surface expression of SIGLEC1 by CRISPR sgRNA activation in WT HeLa cells. **B.** Expression of SIGLEC1 mRNA upregulated by CRISPR sgRNA as measured by RT-qPCR. **C.** cDNA expression in WT HeLa cells. cDNAs encoding the ACE2, SIGLEC1, or SIGLEC1 R116A mutant were overexpressed by lentivirus transduction in WT HeLa cells, and the surface protein expression was analyzed by flow cytometry


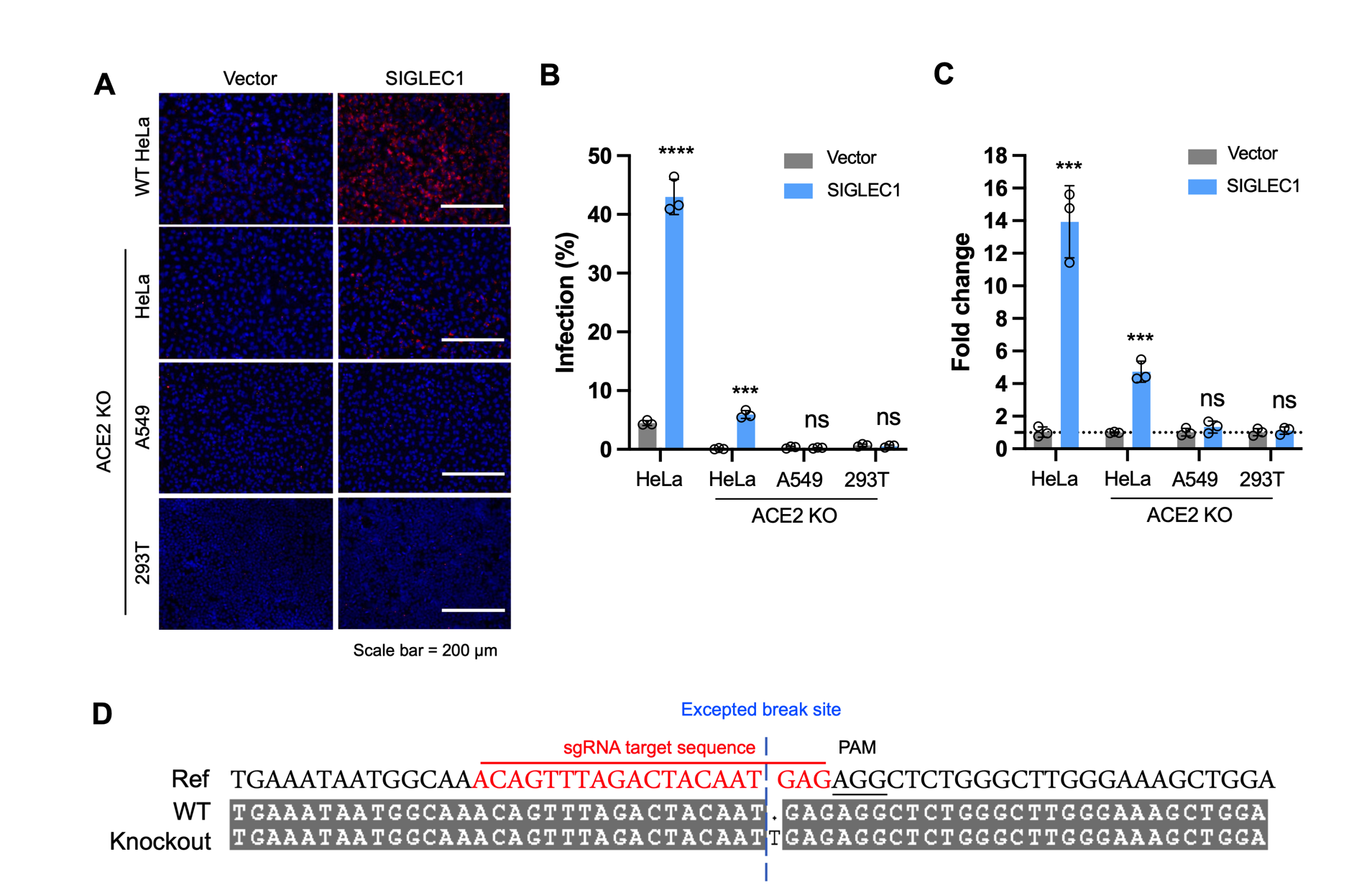
 **Fig.S3. The function of SIGLEC1 in ACE2-deficient cell lines.** A. Representative images of Immunofluorescence staining of N protein upon SARS-CoV-2 infection in WT HeLa, ACE2-KO HeLa, ACE2-KO A549, and ACE2-KO 293T cells. The cell lines were transduced with empty vector or SIGLEC1 cDNA. The images were obtained by using high-content imaging. **B.** Quantification analysis of the high-content imaging of SARS-CoV-2 infection in different cells performed in **A**. **C.** RT-qPCR analysis of SARS-CoV-2 N mRNA upon virus infection in different cell lines. **D.** Schematic of ACE2 gene-editing in both ACE2-KO HeLa and ACE2-KO A549 clonal cell lines. Both cells have the same one insertion of thymine (T) to introduce the frameshifting of translation.


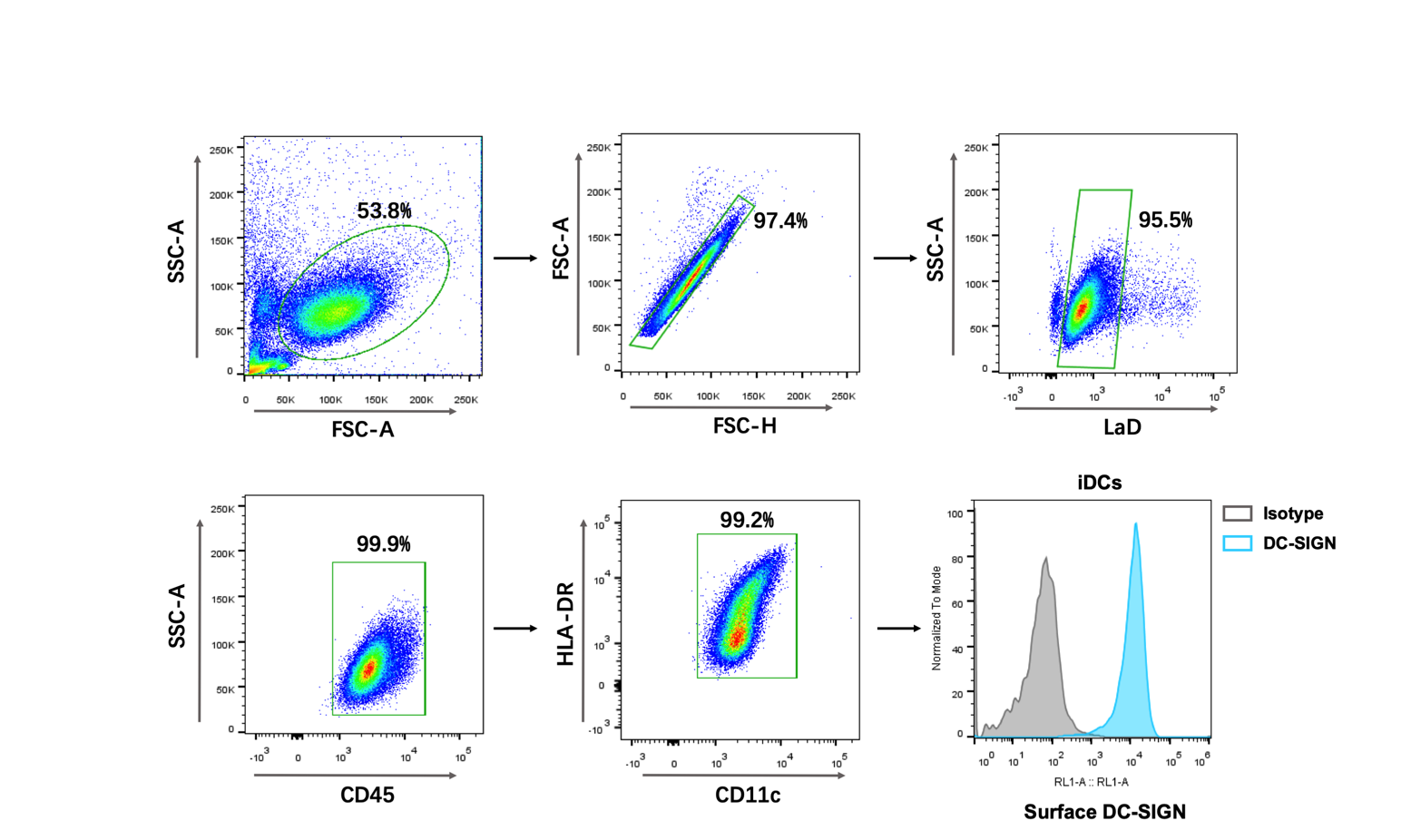


**Fig.S4. Surface expression of SIGLEC1 in differentiated dendritic cells.** **A.** The surface expression of SIGLEC1 in immature DCs, IFN-α- and LPS-DCs. **B.** Surface expression of DC-SIGN in iDCs, IFN-α-DC, and LPS-DCs.


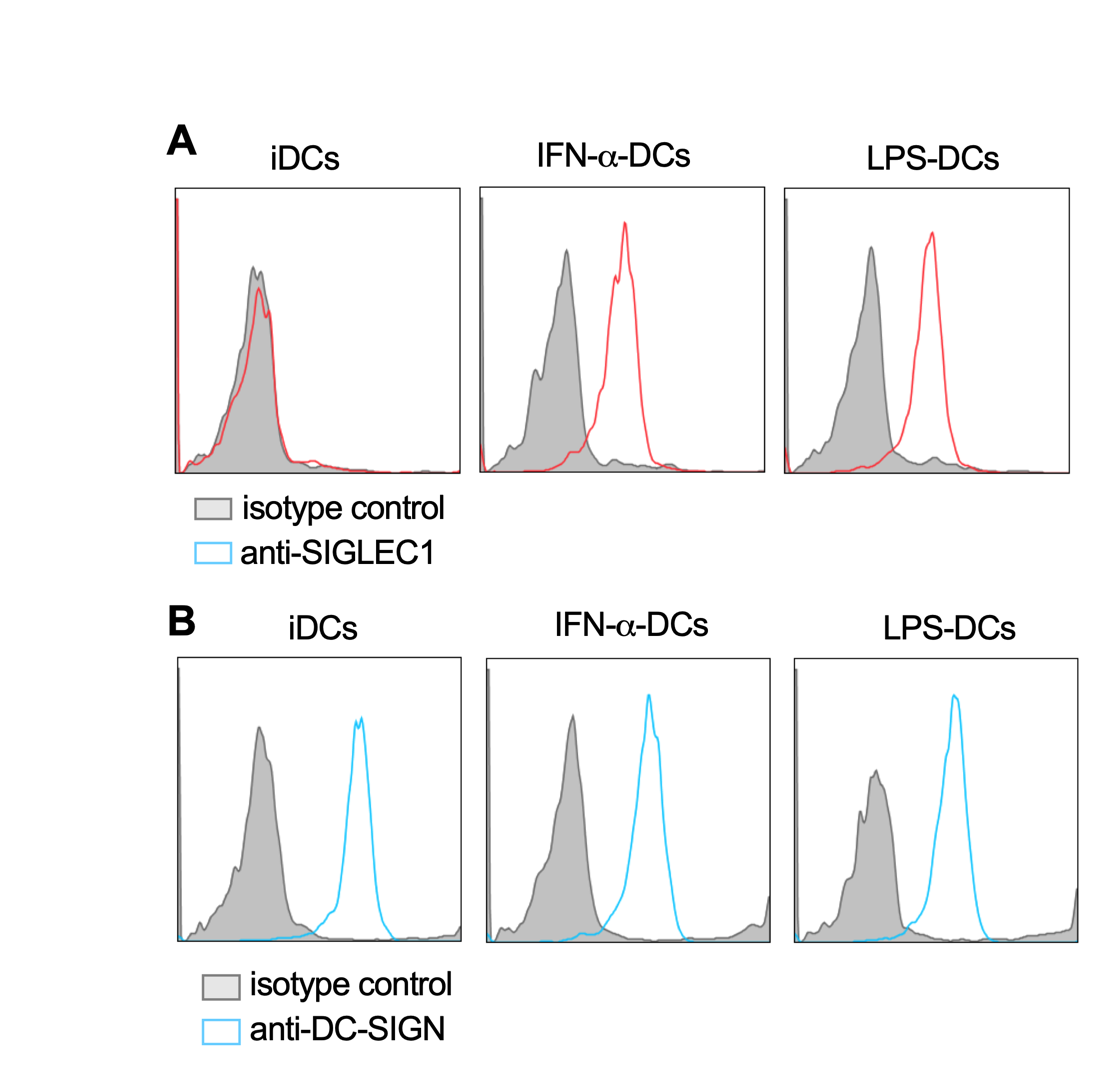


**Fig.S5. The expression markers of monocyte-differentiated iDCs.** Monocyte-differentiated iDCs cells were selected based on unicellularity (singlets/other), viability (live/dead), CD45 expression (CD45+/CD45-), dendritic cell markers expression (CD11c+/HLA-DR+, cell surface DC-SIGN).


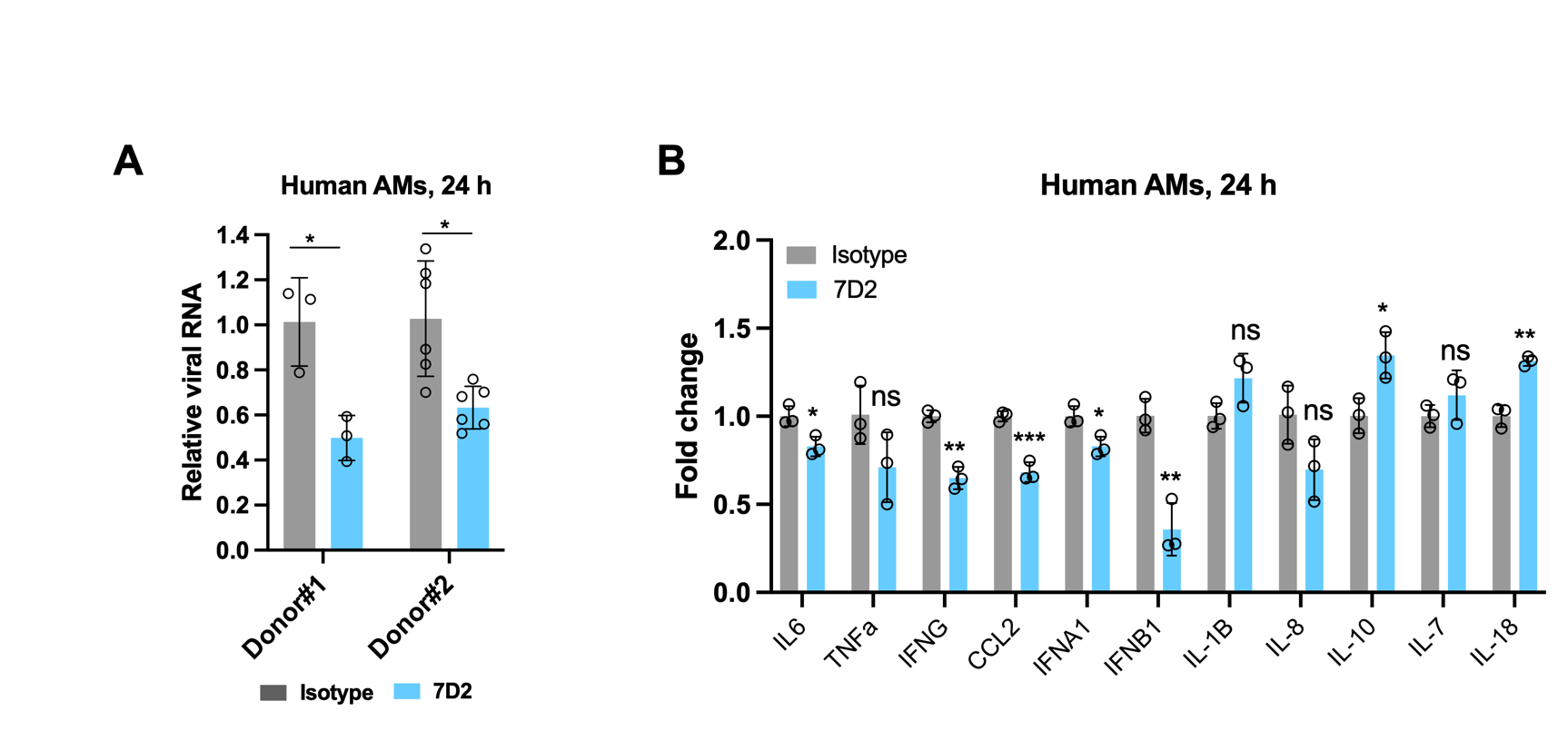


**Fig.S6. The SARS-CoV-2 infection and cytokine production in human primary avelolar macrophages.** The primary alveolar macrophages from human BAL fluid were blocked with isotype or 7D2 antibody followed by SARS-CoV-2 infection for 24 h. The viral RNA (**A**) and cytokine production was determined by qRT-PCR (**B**).
